# Temporal Changes in Electrophysiological Parameters in Untreated Patients With Carpal Tunnel Syndrome

**DOI:** 10.7759/cureus.46039

**Published:** 2023-09-26

**Authors:** Esin Benli Küçük

**Affiliations:** 1 Physical Medicine and Rehabilitation, Niğde Ömer Halisdemir University School of Medicine, Niğde, TUR

**Keywords:** electromyography, entrapment neuropathy, median nerve, electrophysiological examination, carpal tunnel syndrome

## Abstract

Introduction: Carpal tunnel syndrome (CTS) is a common entrapment neuropathy worldwide. This study aimed to investigate the temporal changes in electrophysiological parameters in untreated patients with CTS.

Methods: Patients were recruited among those with the symptoms of CTS who were referred to the electrophysiology laboratory of Niğde Ömer Halisdemir University Bor Physical Therapy and Rehabilitation Hospital in Niğde, Turkey. Forty-nine patients (78 hands) who had not received any sort of treatment for CTS and had prior electrophysiological examination postive for CTS were included. Laboratory records were reviewed retrospectively. Recent electrophysiological parameters of the patients were compared to their prior examinations using Wilcoxon signed-rank test and sign test was used to compare the change in the electrophysiological severity of the study hands between two examinations. One-way analysis of variance (ANOVA) was used to compare individual parameters of the median NCS among electrophysiological change groups (improved, deteriorated, and same).

Results: The mean age was 50 ± 11 years, and 43 (88%) patients were female. The mean duration of time between the two electrophysiological examinations was 37 ± 20 months. Median sensory peak latency and median motor distal latency increased significantly in the second evaluation (p=0.005 and p=0.004, respectively). Median sensory conduction velocity decreased in the second examination (p=0.002). However, CTS severity determined electrophysiologically did not differ significantly in the two examinations (p=0.286).

Conclusion: Although there was a deterioration in electrophysiological parameters during a mean follow-up period of 37 months, the electrophysiological severity of the patients did not worsen.

## Introduction

Carpal tunnel syndrome (CTS) is the most common entrapment neuropathy worldwide [1]. It is caused by the compression of the median nerve at the wrist within the carpal tunnel [2,3]. Symptoms include paraesthesias and dysaesthesias, such as stinging, loss of sensation, or aching in the hand and fingers innervated by the median nerve [1,4]. The frequency and severity of these symptoms may increase with the disease progression. Sensory loss and weakness in the muscles innervated by median nerve can occur. Women are three times more likely to be affected by CTS than men [5]. Clinical history and physical examination together with provocative tests are important in the diagnosis of CTS. Electrophysiological tests are also commonly used to confirm the diagnosis, and these tests provide important quantitative data [2].

The treatment of CTS can be conservative or surgical depending on the severity of the disease [3,6]. Although the favorable effects of these treatment options were widely investigated and reported, a significant number of patients do not use the treatments or are unable to use them for a variety of reasons [7-9]. There are several reports describing the natural course of CTS based on the findings from these untreated patients. Regarding the natural course of the disease, some patients experienced worsening of electrophysiological parameters with time; however, most remained stable over time or improved spontaneously both clinically and electrophysiologically [7,10,11].

To our knowledge, there is no data from our country regarding the course of CTS severity in untreated patients. As lifestyle, hand activities, obesity, genetics, and anthropometric measures have a role in the pathophysiology of CTS, the course of the disease may be expected to differ in various cultures [3,12]. The purpose of the study is to study the temporal change in electrophysiological parameters in CTS patients who have not undergone any treatment and report the natural course of the disease using these parameters.

## Materials and methods

In this retrospective cohort study, the records of patients who were examined in the electrophysiology laboratory of Niğde Ömer Halisdemir University Bor Physical Therapy and Rehabilitation Hospital in Niğde, Turkey, with the symptoms of CTS during the period spanning from January 2019 to February 2020 were reviewed. The study was conducted in accordance with the Declaration of Helsinki. All subjects gave informed consent. The study was approved by the Ethics Committee of the Nigde Omer Halisdemir University.

**Figure 1:**
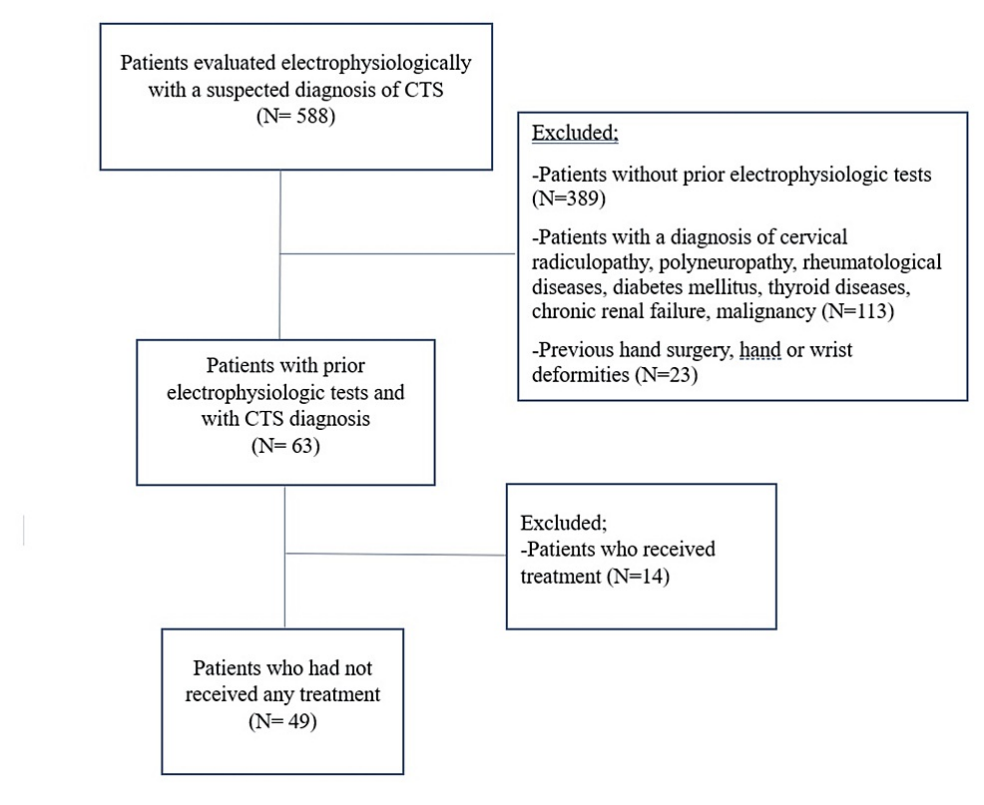
Flowchart of the study CTS: carpal tunnel syndrome

Patients presenting with CTS symptoms and older than 18 years during the examinations were included in the study (Figure 1). Electrophysiological examinations were performed for 588 patients during the study period. Patients with a diagnosis of cervical radiculopathy, polyneuropathy, rheumatological diseases, diabetes mellitus, thyroid diseases, chronic renal failure, malignancy, previous hand surgery, and hand or wrist deformities were excluded. Patients diagnosed with CTS who had been evaluated priorly with electrophysiologic tests in the same electromyography (EMG) laboratory were included. A total of 106 hands of 63 patients diagnosed with CTS were found to be evaluated priorly with electrophysiologic tests. Of the 63 patients, 49 patients who had not received any treatment, including braces, physiotherapy, injections, or surgery for CTS, were included in the study. Electrophysiological examination findings were compared with the findings of previous electrophysiological examinations.

Nerve conduction study

All electrophysiological studies were performed according to the guidelines of the American Association of Electrodiagnostic Medicine (AAEM) [13] for CTS. The Neuro-MEP electroneuromyography (ENMG) tool (Neurosoft Company, Ivanovo, Russia) was used for surface recording and stimulation as described previously [14]. Briefly, in the nerve conduction studies (NCSs), two stainless steel disc electrodes, 1 cm in diameter, were used for recording. Skin temperature was maintained above >33°C throughout the study. The stimulation frequency (1 Hz) and stimulation duration (0.2 msec) were adjusted. Low-pass filters were set to 20 Hz. High-pass filters were adjusted to 10 kHz and 2 kHz for motor and sensory nerve studies, respectively.

For the motor NCS, the stimulation of median and ulnar motor nerves was performed at the level of the wrist, 7-8 cm proximal to the active recording electrode and recorded from the abductor pollicis brevis (APB) muscle for the median nerve and recorded from the abductor digiti minimi muscle for the ulnar nerve. For the sensory NCS, the stimulation of the median and ulnar sensory nerves was performed at digit II and digit V orthodromically at approximately 13-14 cm and 9-10 cm distal to the wrist, respectively.

Normal or pathological measurements were determined using our laboratory normative values. For the median nerve, the conduction velocity of the sensory nerve action potential (SNAP) ≥40 msec and amplitude ≥10 μv were considered normal. For the ulnar nerve, a sensory conduction velocity ≥37.5 msec and SNAP amplitude ≥10 μv were considered normal. Median motor distal latency <4 msec, conduction velocity ≥50 msec, and median nerve compound muscle action potential (CMAP) amplitude ≥4.8 mV were determined as normal. For the ulnar motor, a conduction velocity ≥49 msec was accepted as normal. An ulnar nerve CMAP ≥7 mV was considered normal.

Electrophysiological CTS severity was classified according to the following grades [14-16]: 1) normal (normal findings on all studies); 2) mild CTS (reduced sensory conduction velocity with a normal distal motor latency; 3) moderate CTS (reduced sensory conduction velocity with delayed distal motor latency with a normal CMAP amplitude in the APB muscle); 4) severe CTS (absence of sensory response with delayed distal motor latency or absence of the CMAP amplitude in the APB muscle).

We excluded cases in which no sensory response was observed during NCSs from the statistical analysis that compared the individual electrophysiological parameters of the two electrophysiological examinations. 

Statistical analysis

IBM SPSS Statistics for Windows, version 20 (released 2011; IBM Corp., Armonk, New York, United States) was used for the statistical analysis. Wilcoxon signed-rank test was used to compare the electrophysiological parameters obtained in two examinations. Sign test was used to compare the change in the electrophysiological severity of the study hands between two examinations. One-way analysis of variance. (ANOVA) was used to compare individual parameters of the median NCS among electrophysiological change groups (improved, deteriorated, and same). P values lower than 0.05 were accepted as significant.

## Results

Among the 588 patients evaluated during the study period, 49 patients (78 hands) were included (Figure 1). The mean age was 50.0 ± 10.9 years, and 43 (87.8 %) patients were female. The majority of the patients were housewives (81.6%). Other occupations were office workers (6.1%), laborers (6.1%), and farmers (6.1%).

**Figure 2:**
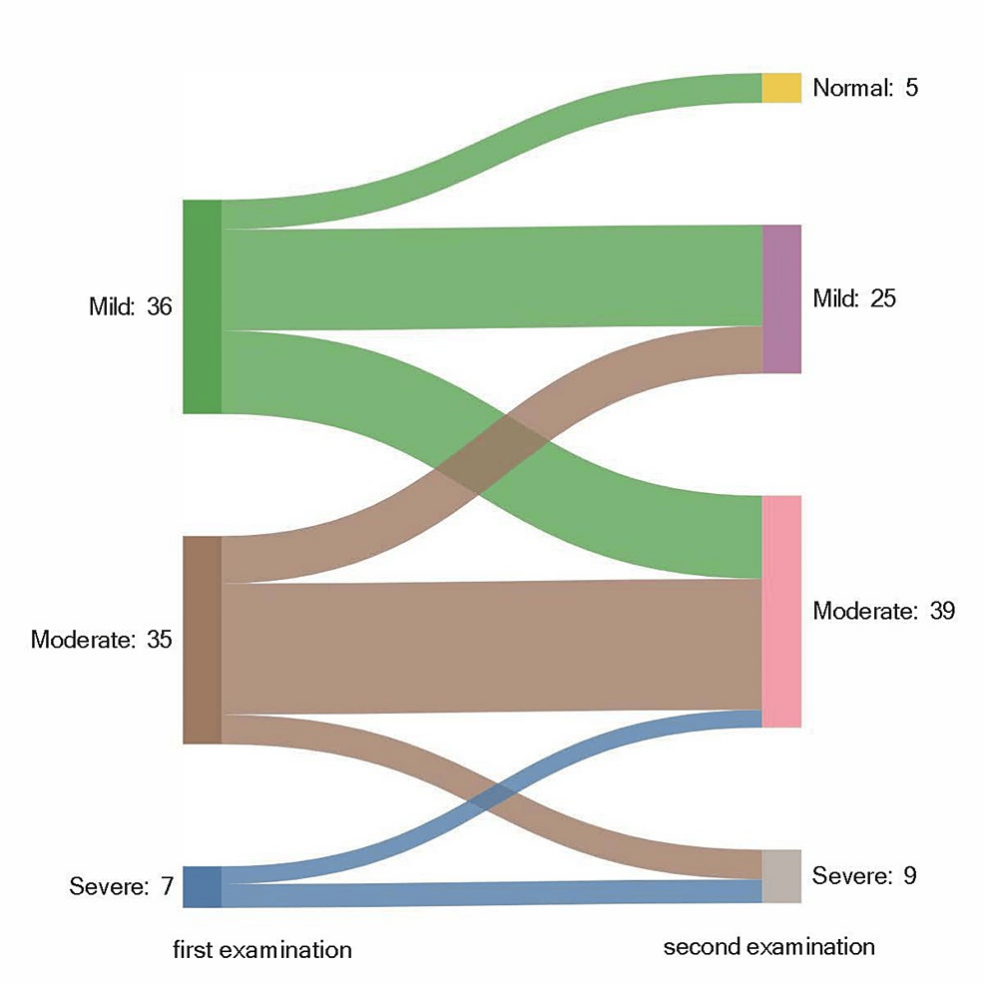
Change in the electrophysiological classification of CTS severity between the first and second examinations

The mean duration of time between the two electrophysiological examinations was 37.2 ± 20 months. Electrophysiological classifications of both examinations are presented in Table 1. The change in the electrophysiological severity of the study hands between the two examinations was not statistically significant (sign test, p=0.286). The change in electrophysiological classification is also presented in Table 2 and Figure 2. Forty-three hands had the same classification, 16 hands improved, and 19 hands showed electrophysiological deterioration.

**Table 1:** Comparison of the electrophysiological classification of 78 hands in the first and second examinations CTS: carpal tunnel syndrome

Severity of CTS	First examination n (%)	Second examination n (%)	p-value
Normal	0 (0%)	5 (6.4%)	p=0.286
Mild	36 (46.2%)	25 (32.1%)	
Moderate	35 (44.9%)	39 (50.0%)	
Severe	7 (9%)	9 (11.5%)	
Total	78 (100%)	78 (100%)	

**Table 2:** Differences in the electrophysiological classification between the first and second electrophysiological examinations

Difference between the first and second examinations	Frequency n (%)
Deteriorated	19 (24.4%)
Same	43 (55.1%)
Improved	16 (20.5%)
Total	78 (100%)

When the patients were grouped in terms of electrophysiological change (improved, deteriorated, and same), there was no significant difference in terms of age or duration of time between two examinations among the three groups (one-way ANOVA, p=0.187 and p=0.953, respectively).

The data regarding the individual parameters of the median NCS are presented in Table 3. Although there was no significant difference in the electrophysiological severity between the exams, there was a change in the individual parameters of the median NCS. The median sensory peak latency and median motor distal latency increased significantly between the exams (Wilcoxon signed-rank test, p=0.005 and p=0.004, respectively). The median sensory conduction velocity decreased, but the median motor conduction velocity did not change significantly (Wilcoxon signed-rank test, p=0.002 and p=0.347, respectively). These individual parameters of the median NCS were not significantly different among the electrophysiological change groups (improved, deteriorated, and same) in the initial electrophysiological examination (one-way ANOVA, for the median sensory peak latency p=0.300, for median sensory conduction velocity p=0.355, for median motor distal latency p=0.240, and for median motor conduction velocity p=0.967).

**Table 3:** Comparison of the electrophysiological findings of the median nerve in the first and second studies *from the wrist to the second digit, **from the wrist to the elbow SD: standard deviation

Electrophysiological parameters of the median nerve	First examination (mean±SD)	Second examination (mean±SD)	p	
Median sensory peak latency* (msec)	4.1 ± 0.7	4.3 ± 0.9	0.005	
Median sensory conduction velocity* (m/sec)	34.0 ± 5.1	32.2 ± 5.8	0.002	
Median motor distal latency (msec)	4.4 ± 1.0	4.6 ± 1.0	0.004	
Median motor conduction velocity** (m/sec)	54.6 ± 5.4	54.2 ± 5.8	0.347	

## Discussion

In this study, we demonstrated that electrophysiological CTS severity did not change despite an increase in the median nerve sensory and motor distal latencies and a decrease in the median nerve sensory velocity in a three-year follow-up of untreated patients with CTS. This is in concordance with previous studies reporting stability and even improvement in NCS in untreated CTS patients [7,11,17,18].

The data about the natural course of CTS is critical to evaluate the effectiveness of the treatments used. Surgery and various conservative approaches were extensively studied in the treatment of CTS [2,19]. However, studies on the natural history of untreated CTS are scarce [16]. These studies report that electrophysiological severity remained stable in most of the untreated patients, and a spontaneous improvement was seen in some. However, the follow-up periods were not very long, usually shorter than two years [7,11,17]. Nathan et al. investigated the median nerve conduction in 289 industry workers longitudinally over 11 years. The mean sensory latencies and prevalence of slow nerve conduction increased, but CTS prevalence did not change [10]. Similarly, we also found that sensory and motor latencies increased, and sensory velocity decreased in three years, but the electrophysiological severity remained relatively stable. Nathan et al. included all workers regardless of symptom presence. However, our study group consisted of patients with symptoms referred to the EMG laboratory.

Ortiz-Corredor et al. followed up 132 CTS patients without treatment for two years [7]. In their study, 7.6% of the patients showed electrophysiological worsening, 67.4% remained constant and 25% improved. In our study, 24.4% of the hands deteriorated, 55.1% remained stable, and 20.5% improved. In another study by Padua et al., 274 hands of 196 patients with idiopathic CTS were prospectively followed up for 10-15 months without treatment. They also reported improvement in electrophysiological severity in 27% of the patients and worsening in 16% of the patients. Electrophysiological severity did not change in 57%. Our results demonstrated lower improvement rates compared to these studies. The longer follow-up in our study, with an average of 37 months, may explain the lower rate of improvement and higher rate of worsening. The variation in the rate of improvement and the likelihood of worsening could also be influenced by different factors, such as lifestyle, hand-related activities, obesity, genetics, and anthropometric measures, all of which play a role in the pathophysiology of CTS and can vary depending on the geographical settings and cultural features. Given that these studies were conducted in different cultural and geographical settings, it is conceivable that differences in the disease's advancement may also arise due to these factors.

Previous studies have reported a high correlation of nerve conduction studies in subsequent examinations and a low correlation of clinical condition in subsequent examinations [7,11]. They stated that these findings support the use of electrophysiological examinations in the diagnoses and monitoring of CTS patients. Electrophysiological evaluations increase diagnostic accuracy in CTS and provide quantitative data to monitor CTS patients [2]. 

There was no difference in age, duration of time between two examinations, and the baseline electrophysiologic parameters among patients with improved, worsened, or similar electrophysiologic CTS severity over time. This finding is in contrast to the findings of Padua et al. who reported more improvement in severe cases and worsening in mild cases. They also reported more frequent improvement in younger patients [11]. This difference may be due to the difference in the study populations and the time between the examinations. We included only NCS confirmed cases, while their study group included cases with normal NCS in addition to cases with abnormal NCS. Moreover, their follow-up period was shorter than that of our study.

There are some limitations of the current study. First, the retrospective nature of the study is a limitation. Second, although the study participants included mild, moderate, and severe cases, the number of severe cases was small. Patients with severe CTS may be more motivated to adhere to a treatment for their condition. Third, we used electrophysiological parameters and classification in our study; we could not use clinical parameters due to the retrospective design of the study. Nevertheless, electrophysiological evaluation provides valuable quantitative data that can objectively represent the status of the median nerve. Moreover, we excluded individuals with secondary causes of CTS, so our results primarily pertain to idiopathic CTS cases. This limits the generalizability of our findings to the broader CTS population.

The long follow-up period is a strength of the current study. Longer follow-up period may increase the probability of detecting subtle temporal changes in electrophysiologic parameters. Another strength of the study is that the electrophysiological examinations were performed by the same physician in the same EMG laboratory. This may increase the accuracy of comparisons performed in this study.

## Conclusions

This study evaluated the temporal changes in electrophysiologic parameters in untreated CTS patients. Although there was a deterioration in electrophysiological parameters during a mean follow-up period of 37 months, electrophysiological severity did not change significantly in the study group. In similar proportions of patients, improvements and deteriorations were observed, but the majority of patients maintained the same level of electrophysiological severity. This finding suggests that conservative methods might be adequate for a substantial portion of CTS patients in the treatment. A deterioration in electrophysiological parameters was noted in our study. Studies with longer follow-up period may reveal whether this deterioration would affect the disease severity in the long term. These results may be helpful to clinicians in counseling patients on suitable treatment options. The findings of this study suggest that conservative methods might be adequate for a substantial portion of CTS patients in the treatment. Utilizing electrophysiological tests for patient monitoring and considering surgical intervention as necessary in cases of progression may be a viable approach. In addition, these results highlight the importance of extended monitoring to assess the potential impact of electrophysiological changes over time.
